# Exploring ART Intake Scenes in a Human Rights-Based Intervention to Improve Adherence: A Randomized Controlled Trial

**DOI:** 10.1007/s10461-012-0175-4

**Published:** 2012-04-20

**Authors:** Cáritas Relva Basso, Ernani Tiaraju Santa Helena, Joselita Maria Magalhães Caraciolo, Vera Paiva, Maria Ines Battistela Nemes

**Affiliations:** 1 Reference and Training Center for Sexually Transmitted Infection and Acquired Immunodeficiency Syndrome, Rua Santa Cruz, 81, Vila Mariana, São Paulo, SP CEP: 4112-000 Brazil; 2 Department of Preventive Medicine, Faculty of Medicine, University of São Paulo, São Paulo, SP Brazil; 3 Regional University of Blumenau, Blumenau, SC Brazil; 4 Department of Social Psychology, Psychology Institute, University of São Paulo, São Paulo, SP Brazil

**Keywords:** Antiretroviral therapy, HAART, HIV/AIDS, Adherence intervention, Psychosocial intervention, Social constructionism, Human rights

## Abstract

To assess the effectiveness of a psychosocial individual intervention to improve adherence to ART in a Brazilian reference-center, consenting PLHIV with viral load >50 copies/ml were selected. After 4 weeks of MEMS cap use, participants were randomized into an intervention group (IG) (*n* = 64) or control group (CG) (*n* = 57). CG received usual care only. The IG participated in a human rights-based intervention approach entailing four dialogical meetings focused on medication intake scenes. Comparison between IG and CG revealed no statistically significant difference in adherence measured at weeks 8, 12, 16, 20 and 24. Viral load (VL) decreased in both groups (*p* < 0.0001) with no significant difference between study groups. The lower number of eligible patients than expected underpowered the study. Ongoing qualitative analysis should provide deeper understanding of the trial results. NIH Clinical Trials: NCTOO716040.

## Resumen

Personas viviendo con VIH con carga viral >50 copias/ml fueron seleccionadas en un centro de referencia en Brasil para evaluar la efectividad de una intervención psicosocial individual para mejorar la adhesión a la terapia antirretroviral. Los participantes firmaron el documento de consentimiento informado. Tras 4 semanas de uso de MEMS caps, los participantes fueron aleatorizados en el grupo de intervención (GI) (*n* = 64) o el grupo control (GC) (*n* = 57). El CG recibió solamente los cuidados habituales. El GI participó de una intervención basada en los derechos humanos, con cuatro reuniones dialógicas centradas en escenas de toma de medicamentos. La comparación entre GI y CG no reveló ninguna diferencia estadísticamente significativa en la adhesión medida en las semanas 8, 12, 16, 20 y 24. La carga viral (CV) disminuyó en ambos grupos del estudio (*p* < 0.0001), aunque sin significado estadístico. El número de pacientes elegibles, más pequeño del que el esperado, disminuyó el poder del estudio. El análisis cualitativo actual debe proporcionar una comprensión más profunda de los resultados del estudio. Registro del Ensayo Clínico: NCT00716040.

## Introduction

Successful antiretroviral therapy outcomes depend on high patient adherence to the treatment [1, 2], although recent reports have demonstrated that moderate levels of adherence can also promote viral suppression [3]. Low levels of adherence have been shown to increase risk of disease progression burden on services and treatment cost [4–8]. Non-adherence facilitates the emergence of resistant strains of the virus (limiting future treatment options) and provokes spread of the disease among the community [9]. Adherence has become crucial to AIDS programs worldwide [10–12] and has led to the implementation of interventions aimed at enhancing adherence [13].

In Brazil, besides universal and free access to health care and antiretroviral therapy, all PLHIV are also entitled to free access to CD4, Viral Load and Genotyping exams. The National AIDS Program has provided general guidance to the health system regarding adherence [14].

As analyzed by Caraciolo [15], most AIDS care services report that adherence is assessed and encouraged, mainly during clinical consultations with physicians and nurses. Additionally, services carry out different initiatives to enhance adherence, such as group sessions or conversations and talks in the waiting room. Adherence promotion activities however, differ greatly across local health services, and are rarely evaluated. Moreover, most these activities are aimed at all patients and there are no reports of interventions aimed specifically at non-adherent patients. Few studies have been performed in Brazil investigating the efficacy of these interventions [16, 17], and only one randomized controlled study has been published to date [18].

Considering this context, University of São Paulo researchers established a partnership with the São Paulo AIDS Program to conduct a broader project to develop, implement and analyze a psychosocial intervention and its acceptability, feasibility and effectiveness within the Brazilian Unified Health System (SUS).

The intervention drew on the references which have distinguished the ethical and theoretical framework underlying the Brazilian Response to AIDS (BRA) [19, 20], more specifically, its human rights-based approach [21–23]. Non-discrimination and participation are core components of the right to health, and of human rights-based responses to AIDS, as well as the principles of availability, accessibility, acceptability and quality of the services delivered, ensuring the most vulnerable populations are reached with the services they need [24]. In a debate published in the *Lancet*, Kalichman and Diniz [25] noted that mortality and incidence decreased following this rights-based approach providing the necessary evidence. BRA would have never succeeded without the SUS principle of universalidade (universal access), grounded in constitutional rights for both prevention and treatment.^1^ A common misunderstanding centers on the Unified Health System (SUS) principle of integralidade (comprehensiveness) aimed to overcome the dichotomy between prevention and treatment.

For two decades, *integralidade* and *universalidade* have been key assets to the quality of services delivered and for the high rates of adherence to ARV in Brazil, as previous studies have shown [26]. Early in the 1990s, the National Aids Program defended before the World Bank that treatment is in itself a form of secondary prevention of complications, reduces HIV transmission and bring affected people to the health system for testing, counseling and early treatment [19, 25]. On the other hand, much of what was learnt in BRA prevention policies focused on universal access and adherence to condom use inspired the integralidade initiatives enhancement of AIDS care and treatment [27].

Two characteristics of the intervention discussed in this article are consistent with features of effective interventions analyzed in international systematic reviews, namely, the fact that it is an individual approach (compared to a group approach) [28] and is aimed at non-adherent patients [29]. It is also congruent with approaches that propose a more radical replacement of the notions of adherence with ideas that reflect “collaboration” [30], “autonomy motivation” [31], “empowerment” [32] and the notion of “concordance” [33], as we have discussed elsewhere [30].

This paper discusses the results of a clinical trial that evaluated the effectiveness of a psychosocial intervention developed from previous BRA initiatives that conceptualize health care as inextricably linked to prevention [34–36], especially based on the concept of Care (Cuidado) [30, 37] and its psychosocial constructionist approaches [38–40].

## Objective

The aim of this study was to evaluate the effectiveness of a social-psychological individual intervention for improving adherence to antiretroviral drug regimens for HIV.

## Methods

### Study Setting

This study was conducted in 2008, from March to November, at the STI/AIDS Training and Reference Center of Sao Paulo State, Brazil (CRT-DST/AIDS), a traditional “gold standard” reference center for *integralidade* and interdisciplinary approaches for the Brazilian Aids Response (BRA), which has provided more than two decades of care. At that present time assisted over 4,000 PLHIV, attracting patients coming from different parts of the metropolitan area, from other cities and regions of the country. The CRT-DST/AIDS has been an operational research center for SUS. It has permanent multi-disciplinary forums comprising nurses, psychologists, social workers and dental surgeons, infectious disease physicians and other specialists (psychiatrist, neurologist, cardiologist, endocrinologist, urologist, proctologist, dermatologist, etc.).

### Participants

Clients of the service older than 18 years of age with a blood-detectable HIV viral load of more than 50 copies/ml and undergoing treatment with the same antiretroviral regimen for at least six months prior to the date of viral load results from exams done at recruitment, were invited to take part in this study (preliminary criteria). A detectable Viral Load after 6 months in use of HAART in the absence of proven viral resistance, strongly suggests an adherence problem [41]. The following exclusion criteria were applied: a) pregnancy (adherence issues are diverse and specific); b) having a physically or mentally disabling disease which prevents individual from visiting the service or taking part in the proposed activities; c) in treatment for hepatitis B or C, or for active opportunistic disease (to prevent accumulation of other types of medication); d) previous inclusion in any other clinical trials, a requirement for any research at the Center.

### Sample Size

The sample size was calculated to detect a difference in the average adherence (primary study measure) of 20 % between the Intervention and Control Groups at the end of study, considering 30 % of estimated average adherence in the Control Group, with a confidence interval of 95 %, power (1−β = 0.80 and α error = 0.05). Sample size was estimated at 100 subjects per group, allowing for 10 % refusal. A post hoc power analysis was performed to evaluate the primary study measures of adherence and also to at 1 and 6 months.

### Recruitment and Allocation

A list of adult patients who met the preliminary eligibility criteria were contacted briefly at their appointment by a member of the research team who invited them to an interview. Those who did not have an appointment during the recruitment period were contacted by phone. In the pre-scheduled interview, a nurse from the research team assessed the exclusion criteria and potential subjects were informed about the aims of the study, its duration, intervention and procedures. Those who agreed to participate proceeded to the informed consent process. Participants were, then, randomized at a 1:1 ratio and allocated into either the Intervention Group (IG) or the Control Group (CG).

A computer-randomized number list was produced by an independent statistician and kept under lock and key at the Research Unit (RU) of the CRT/DST/AIDS in accordance with its ethical procedures. The allocation was carried out after the baseline interview when the nurse contacted the person in charge of the computer-randomized list at the RU by phone, informing the patient ID number. The nurse was then furnished with the allocation (IG or CG) according to the list sequence. The study procedures were approved by the CRT-DST/AIDS review board, as required by the National Ethics Committee of the Brazilian Ministry of Health.

### Intervention Description

The structure of the intervention (Table 1) consisted of four individual 1 hour meetings held every fifteen days by previously trained health professionals according to the following guidelines.

**Table 1:** Summary of objectives and structure of ACCA* intervention

	Session 1	Session 2 e 3	Session 4
Objectives	Contract;	Increase knowledge about treatment;	Deeper understanding of feasible and desired changes in context and personal conduct aiming at self-care and enhancement of patient-clinic quality of communication and care;
	Identify situations ant context of daily life that are obstacles for treatment;	Understand and *decodify* real life scenes;	Identify resources to pursue and sustain chosen paths to face difficulties with ARV treatment;
	Organize priority issues and decide on themes to be part of next conversations;	Amplify daily scene to bigger programmatic and social context;	Close the process.
	Clarify most technical question about treatment.	Foster creative and active imagination about daily life	
		Foster new personal repertoires to face identified obstacles to treatment.	
Themes	Mutual recognition of patient as experts on daily life and professionals-researchers as technical experts;	Questions about treatment;	Questions about treatment;
	The overview of patients’ social and inter-subjective context;	Real episodes where treatment is not followed;	Reviewing paths, solutions and repertoires;
	Question about treatment.	Paths to face obstacle and “in scene” solutions.	Talking about how to face future obstacle and difficulties and sustain changes;
			Final clarification and orientation on the research process.
Methodology	Talking about the procedure, aim and contract;	Reviewing contract and questions;	Reviewing contract and questions;
	Free conversation and careful listening about the person’s life	Looking at typical episodes of non-adherence	Taking and exploring scene from real episodes;
	Focus questions about treatment and on situations and episodes where following treatment is difficult;	The participant chosen their priority from list of problems;	Decoding the scenes, and through active imagination and role-playing reinvent them;
	Use of informative resources(folders, guidelines, adherence kits);	Taking and exploring scenes from real episodes;	Inform on social and programmatic resources, as well as constitutional rights
	Records specific situations and episodes that seem to be more important to cope on recording sheets	Decoding the scenes, and through active imagination and role-playing reinvent them;	Constitutional rights; Recording decisions and plans for the future on recording sheets.
		Talking about obstacle that are beyond individual action, and shared by other PLHIV;	
		Discussing individual and programmatic resources;	
		Professional and participant record and organizing a hierarchy of scenes and situations on *recording sheet*	

* ACCA is the acronym for “Abordagem Construcionista do Cuidado em Adesã”(Constructionist Care Approach to Adherence)

#### Goal

The intervention was conceived as Cuidado (Care), a process aimed at “technical success” (good clinical outcome). Technical success depends “on practical success, i.e., the ability of health care to focus, beyond the clinical outcome, on the health-related aspirations of patients, and should include a negotiation of the best possible outcome given the patient’s life plan and well-being at the time” [30]. Practical success constitutes greater convenience for the patients based on their own definition of a “good life” and is a good marker of the human rights principle of non-discrimination and acceptability.

#### Method

Decoding and overcoming limiting situations shall be defined as the core of a shared process of collaboration towards Cuidado, based on professional-patient mutual recognition, conversation and dialogue. The patient is conceived as the expert on their daily life whereas the professional is conceived as the expert on the technical side of medical practice and health promotion [40].

Dialogue, a good marker if participation that enhance quality of care, should focus on daily routine “scenes” that expressed (coding) real experiences of ARV and medication intake. Conversation should reach the deepest understanding of each inter-subjective context dynamic of the intake scene, amplified to the comprehension of social, cultural and structural scenery [38–40]. The dialogue focused on the “person in scene” mirrors the “coding–decoding” process as proposed by Paulo Freire [42] pedagogic constructionist approach; it is also based on social constructionist dramaturgical approaches to human interaction applied to the AIDS responses, which consider gender and sexuality inequalities as well as processes of stigmatization and discrimination [40, 43–48].

#### Ethical Horizon

Patient individuality and autonomy must be respected, with patients recognized as having rights and being entitled to the constitutional right to non-discrimination and to universal, *integral* (comprehensive) care, treatment and prevention.

Close supervision of researchers—nurses, psychologists and social workers with previous experience in AIDS Reference Centers—was carried out based on recording sheets and the personal reports produced by them. All meetings were tape-recorded.

In this constructionist approach, the professional acts as a “director” of an “imaginary drama”. Conversations look for real episode narratives focusing on scripts and characters in action, the ARV intake scene dynamics in this case. Lists of problems, behaviors, attitudes, believes, knowledge, motivations and emotions are approached through real life scenes, chosen by the professional. Scenes are conceived as embodied scripts, situated in the broader social context where gender, social status and power imbalances are constructed. Without leaving the typical two-chairs-one-table ambulatory setting, spontaneity is increased as the professional explores positions and role changes by requesting the patient to be, for a moment, in the other person’s shoes or to change their own scripts, supporting them to actively re-invent the scene. The deeper and shared understanding of the daily medication intake obstacle dynamic occurs when the “medication taker” can be seen in their role, as well as the roles of “others”, and can see their scenery in a clearer and fuller manner, by exploring scene meanings in the capacity of spectator from an outside perspective, while using active imagination to test new performances and scenes. Patient and professional conclude by exploring viable new plans of action. Beyond sharing the deeper understanding of daily life, professional technical knowledge and practical experience of the person in scene, which includes learning from situations experienced by other patients, are shared as testimonies of professional expertise.

### Usual Care

Participants from the control group received only usual care. Both intervention and control group participants attended routine consultations with their assisting physician scheduled every 2 months, or more frequently when clinically indicated. The medical consultations lasted 40 min on average. A reference team that comprised a physician, psychologist and social worker saw all participants. Adherence was approached in a range of different initiatives. Specifically, the physicians and nurses investigated the use of medication in accordance with the prescription, difficulties related to medication use and adverse effects, and sought to adjust the timing of medications to suit the daily routine of the patients. Medical specialists saw participants when necessary.

All participants had an appointment with nurses who read the MEMS caps (Medication Event Monitoring System, AARDEX, Ltd., Zug, Switzerland) and assessed how they were coping with this tool. The nurses also encouraged adherence to the study, representing important input to the intervention process.

### Outcome Measures

#### Adherence Measures

MEMS caps were used as an electronic monitoring device. Subjects received their medicines from the pharmacy in bottles with MEMS caps, and after two months of follow-up were randomized to receive intervention or standard care. Two medicines were monitored independently of the antiretroviral regimen. If antiretroviral regimen was more complex (more than two drugs), the drug with the highest number of pills or frequency of doses or adverse effects, was chosen.

Participants returned to collect their medicines monthly and their adherence was measured using the electronic monitoring device at weeks 8 (pre intervention), 12, 16 (intervention period), 20 and 24 (post intervention period).

The adherence measure was estimated based on percentage of doses taken (total dose taken divided by total doses prescribed multiplied by 100), percentage of doses taken on time (accepted variation tolerance of up to 25 % above or below) and according to the proportion of individuals who took 95 % or more of doses prescribed.

#### Viral Load

HIV1 RNA levels were assessed by VERSANT-HIV-1 RNA 3.0 b-DNA Essay, detection limits = 50 copies/ml—Bayer Health Care—b-DNA Analyzer System 340 in the CRT/DST/AIDS laboratory. Viral load expressed in logs was measured both at baseline and at the end of the study.

### Statistical Analysis

The analysis was done through intention-to-treat. Groups were first compared for covariables obtained at baseline in order to verify any differences at time of inclusion that could have potentially interfered with outcomes. Results were analyzed by comparing means (Student’s *t* test) and proportions (Pearson’s Chi-square test) of outcome variables between CG and IGs. During follow up, variations in mean outcomes at study baseline were compared against subsequent measurements using Student’s paired *t* test for means, and McNemar’s test for proportions. Kruskal–Wallis and Wilcoxon’s non-parametric tests were applied when the distribution type of the study variables did not have a normal distribution. Linear regression was also calculated for proportion of individuals with adherence of greater than or equal to 95 % by groups (normality and homoscedasticity was examined) at baseline, and at 30, 60 and 90 days’ follow-up, using the coefficient of straight-line angle and coefficient of determination (*r* ^2^). The level of statistical significance was set at a value of *p* < 0.05.

## Results

Of a total of 566 patients who had viral load exams performed within four months of study commencement, 363 eligible subjects were identified and underwent the recruitment process between 13th March and 28th May, 2008. Of the eligible group, 121 agreed and 240 refused to take part in the study while two individuals were excluded (1 pregnancy and 1 presenting with active opportunistic disease). Among refusal reasons, the need to attend the service more frequently than routine care was indicated by 18 % of participants. Fifteen percent did not show up after first contact and other 8 % argued that they lived in another city. The remaining refusal motives were scattered.

**Fig. 1:**
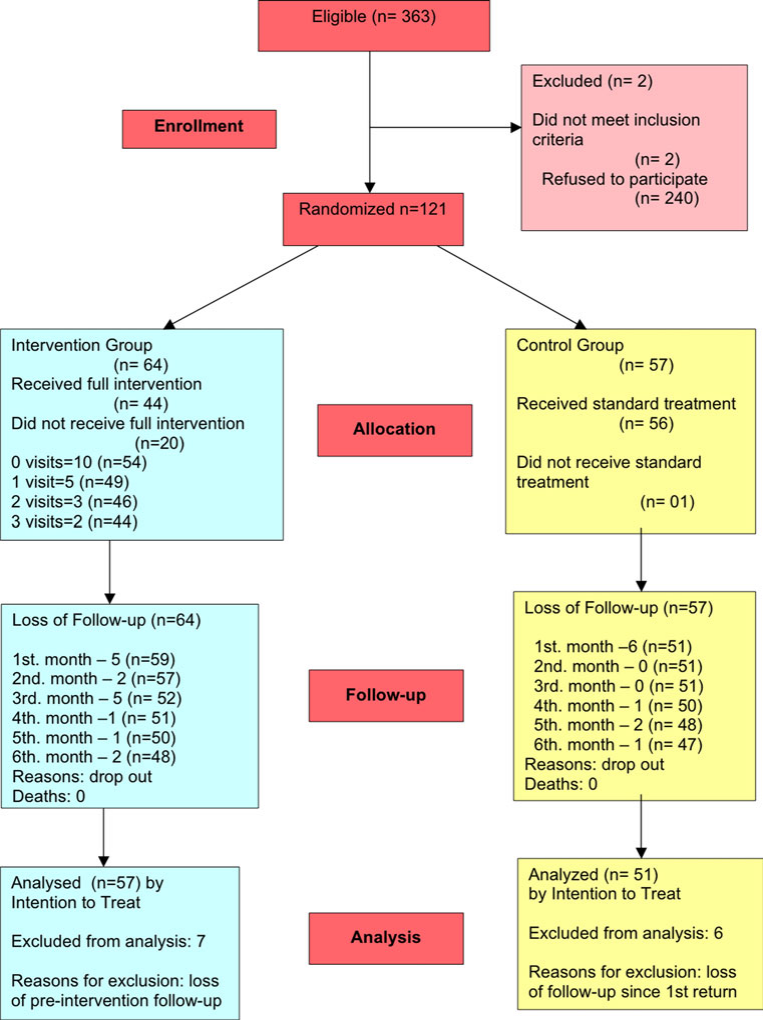
Flow diagram of patients’ progress throughout the study

The enrollment, group allocation, follow-up and data analysis are depicted in the diagram constructed according to CONSORT guidelines (Fig. 1). No statistically significant difference between the IG and the CG was found for the variables collected in the beginning of the study, as shown in Table 2.

**Table 2:** Socio-demographic and epidemiological characteristic of participants at study baseline

Variable	Intervention (*n* = 64)	Control (*n* = 57)	*p* value
Sex, n (%)			
Male	40 (62.5)	36 (63.2)	0.94^a^
Age (years)			
Mean ± SD	42.8 ± 7.7	42.9 ± 8.6	0.97^b^
Schooling, n (%)			
Primary	29 (45.3)	20 (35.1)	0,47^a^
Secondary	22 (34.4)	22 (34.4)	
Higher	13 (20.3)	12 (21.1)	
Time with HIV (months)			
Mean ± SD	134.7 ± 63.8	144.1 ± 57.7	0.39^b^
Time under ART (months)			
Mean ± SD	99.8 ± 50.0	105.4 ± 44.2	0.52^b^
Adverse Effects, n (%)			
Yes	28 (43.8)	29 (50.9)	0.43^a^
Viral load (Log)			
Mean ± SD	3.4601 ± 1.1967	3.3046 ± 1.0944	0.47^b^
Smoking, n (%)	32 (50.0)	21 (36.8)	0.15^a^
Drugs use, n (%)			
Alcohol consumption^c^			0.34^a^
Less than once a month	47 (74.6)	38 (66.7)	
Less than once a week	4 (6.4)	2 (3.5)	
Weekly or more	12 (19.0)	17 (29.8 %)	
Cannabis^c^			0.08^a^
Less than once a month	57 (91.9)	44 (78.6)	
Less than once a week	0 (0.0)	2 (3.5)	
Weekly or more	5 (8.1)	10 (17.9)	
Cocaine^c^			
Less than once a month	62 (98.4)	50 (87.7)	0.06^a^
Less than once a week	0 (0.0)	1 (1.8)	
Weekly or more	1 (1.6)	6 (10.5)	
Common mental disorders, n (%)	48 (75.0)	45 (79.0)	0.61^a^

^a^Chi-square test

^b^ *t* student test

^c^missing or excluded

Mean time in the study was similar for both groups (IG 159.1 days SD = 67.0 vs. CG 165.1 days SD = 61.361, *t* test = 0.514; *p* = 0.61). Frequency of visits to the service for other activities was also similar for both groups (IG 8.8 SD = 5.4 vs. CG 8.3 SD = 5.2, *t* test = 0.517; *p* = 0.61). The mean interval between study intervention visits was 21 days.

The overall retention rate was 83.4 % (79.6 % in the IG, and 89.4 % in the CG) at the end of the intervention. Forty-four participants attended all four meetings, corresponding to 69 % of the participants randomized into the IG. Retention was 80.9 % (78.1 % in IG, and 84.2 % in CG) at the first follow up after the end of intervention, and 78.5 % (75 % in IG and 82.4 % in the CG) at the end of the study.

During the first two months of use of the electronic monitoring device, the percentage of adherence for doses taken ranged from 85.9 (1st month) to 78.0 % (2nd month) in the IG, and from 82.3 (1st month) to 77.5 % (2nd month) in the CG, with no significant differences. The proportion of adherent participants (95 % adherence or greater) ranged from 50.9 (1st month) to 36.8 % (2nd month) in the IG, and from 49 (1st month) to 50.9 % (2nd month) in the CG. The adherence values for the second month were taken as the baseline measures prior the Intervention.

No statistically significant differences were found between the adherence percentages for doses taken in the IG and the CG for the five measurements obtained, as shown in Table 3. Considering this measure, the power at the end of the study was 3.5 %. However, the fall in adherence in the IG observed at the second follow up (120 days) was statistically significant (Wilcoxon test *z* = 2.251; *p* = 0.02). No falls in adherence percentages were found for the CG at the follow up periods.

**Table 3:** Adherence percentages for doses taken, measured by MESM cap,from commencement of intervention, by group

Measurement intervals	Intervention x ± SD	Control x ± SD	*p* value*
Commencement of intervention	78.03 ± 29.92 (*n* = 57)	77.45 ± 31.52 (*n* = 51)	0.60
After 30 days	81.48 ± 34.38 (*n* = 52)	80.13 ± 28.40 (*n* = 51)	0.78
After 60 days	79.21 ± 31.49 (*n* = 51)	79.06 ± 29.64 (*n* = 50)	0.86
After 90 days (first follow-up)	76.99 ± 37.70 (*n* = 50)	79.38 ± 31.25 (*n* = 48)	0.80
After 120 days (second follow-up)	74.82 ± 32.97 (*n* = 48)	76.26 ± 34.54 (*n* = 47)	0.49

* Kruskal–wallis test with 1 d.f.; all *p* > 0.05

No statistically significant difference was found between the adherence percentages for taking medication according to the prescribed time regimen in the IG and the CG, for the five measurements obtained, as shown in Table 4. Considering this measure, the power of the study was 12.0 %. The fall in adherence in the IG at the first and second follow ups (90 and 120 days) was statistically significant (Wilcoxon test *z* = 2.535; *p* = 0.01 and *z* = 2.805; *p* = 0.005, respectively). In the CG, no falls in adherence percentages were found at the follow up periods.

**Table 4:** Adherence percentages for doses taken at prescribed time, measured by MESM cap, from commencement of intervention, by group

Measurement intervals	Intervention x ± SD	Control x ± SD	*p* value*
Commencement of intervention	55.93 ± 30.61 (*n* = 57)	61.9 ± 35.9 (*n* = 51)	0.21
After 30 days	56.63 ± 33.87 (*n* = 52)	59.16 ± 34.18 (*n* = 51)	0.72
After 60 days	60.55 ± 32.51 (*n* = 51)	59.29 ± 33.51 (*n* = 50)	0.84
After 90 days (first follow-up)	51.54 ± 36.75 (*n* = 50)	57.07 ± 35.20 (*n* = 548)	0.49
After 120 days (second follow-up)	47.06 ± 34.24 (*n* = 48)	57.55 ± 36.03 (*n* = 47)	0.11

* Kruskal–wallis test with 1 d.f.; all *p* > 0.05

No statistically significant difference was found between the proportion of patients with adherence greater than or equal to 95 % of doses taken in the IG and the CG, for the five measurements obtained, as shown in Table 5. Considering this measure, the power at the end of the study was 9.9 %. In the IG, a fall in the proportion of adherents was seen at the second follow up (120 days) but did not reach statistical significance (Exact test value of *p* = 0.09). No falls in adherence percentages were found for CG at the follow-up periods.

**Table 5:** Proportion of patient with adherence percentages greater than or equal to 95 %, measured by MEMS cap

Measurement intervals	Intervention (95 % CI)	Control (95 %CI)	*p* value*
Commencement of intervention	36.8(24.4–50.7) (*n* = 57)	50.9(36.6–65.2) (*n* = 51)	0.14
After 30 days	50.0(35.8–64.2) (*n* = 52)	47.1(32.9–61.5) (*n* = 51)	0.77
After 60 days	47.1(32.9–61.5) (*n* = 51)	44.0(30.0–58.7) (*n* = 50)	0.76
After 90 days (first follow-up)	50.0(35.5–64.5) (*n* = 50)	45.8(31.4–60.8) (*n* = 48)	0.68
After 120 days (second follow-up)	35.4(22.2–50.5) (*n* = 48)	44.7(30.2–59.9) (*n* = 47)	0.36

* Chi Square test 1 d.f.: all *p* > 0.05

**Fig. 2:**
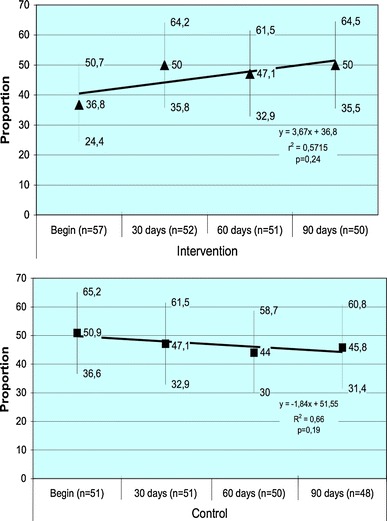
Linear regression of proportion of patient’s adherence greater than 95 % by group (Intervention and control)

Figure 2 shows the linear regression for the proportion of individuals with adherence greater than or equal to 95 % (95 % Confidence Interval) at the commencement of the intervention and at 30, 60 and 90 days of follow up, by group (Intervention and Control).

With regard to the secondary outcome measurement, only 80 volunteers had viral load exams available at the end of study. Among these 80 participants, there was no significant difference between the IG and the CG in terms of the proportion of patients with detectable viral load at the beginning of the study that evolved to undetectable viral load at the end of the study.

We also recalculated the initial viral load for these patients, yielding a result of 3.2573 Log in the IG and 3.2689 Log in the CG. No statistically significant difference in final viral load values was found between the Intervention (*n* = 44) and Control (*n* = 36) Groups, 2.4390 ± 1.4611 Log versus 2.3086 ± 1.3567 Log, respectively, (*t* test = 0,4100; *p* = 0.68). Nevertheless, a slight decrease in viral load was observed between study baseline and endpoint in both groups, Intervention 3.2573 Log versus 2.4390 Log (Paired *t* test = 3.484; *p* < 0.01) and Control 3.2689 Log versus 2.3086 Log (Paired *t* test = 4.332; *p* < 0.0001).

The fall in viral load was greater in study participants compared to those who refused to take part in the study. The viral load at the end of study was 2.3803 Log in volunteers and 2.8536 Log in refusers, with a value of *p* < 0.05.

## Discussion

*Baseline measures* of adherence rate—86 % in the IG and 81.6 % in the CG—were higher than expected. Furthermore, electronic monitoring typically achieves lower adherence than pill counting while pill counting attains lower adherence than self-reports [49, 50]. The Hawthorne effect [51], willingness to participate, and MEMS caps use for two months prior to the intervention may have previously increased mean adherence. The fall in adherence between the first and second month, when supposedly MEMS caps was an easier routine for participants, may be evidence of this effect.

The increased care given to the patients during the study period, which led to more visits to the service as well as differentiated attention provided to both Control and Intervention groups, may have contributed to increased adherence to the anti-retroviral therapy by all participants involved in this study. Other uncontrolled factors present in this study may have promoted a strong response in the CG, thereby minimizing any differences [52].

The adherence percentages according to doses taken and to time regimen prescribed, as well as the proportion of patients with adherence of greater than or equal to 95 %, did not differ significantly between the IG and CG.

The proportion of patients with adherence greater than or equal to 95 % during the intervention period and the first follow-up period however, showed a tendency toward increased adherence in the IG, and reduced adherence in the CG, although this did not reach statistical significance (Fig. 2). The decrease in adherence observed after the end of the intervention solely in the IG presented statistical significance, and supports the hypothesis that this may have stemmed from the effects of the intervention, given that both groups were equally exposed to the effect of MEMS. Other studies have shown that adherence levels do not persist after the intervention, tending to decline over time [53, 54].

The increases in adherence promoted by complex interventions, even the most effective of these interventions, has proved to be limited [55]. Simoni et al. [56]., in a recent meta-analysis of randomized controlled trials which assessed the impact of stimulus interventions on adherence to antiretroviral therapy, noted a 1.5-fold likelihood of intervention participants attaining 95 % adherence. To date, there is no evidence that low adherence can be remedied in a definitive manner. Therefore, strategies to enhance adherence should be continued throughout the treatment period [55] and include periodical actions aimed at enhancing adherence, particularly among individuals who struggle to manage their treatment [29, 53].

The sole previous randomized controlled study of an intervention to enhance adherence to ARV conducted in Brazil [18] was held in the State of Bahia and used self-reports to measure adherence. Results also showed no effect of the intervention on adherence. Van Dulmen et al. [57]. analyzed 38 systematic reviews focused on chronic disease adherence interventions and revealed that half of these had no effect on adherence.

*Measurements of secondary outcomes* also showed no effect when comparing viral load between IG and CG. However, the fall detected in both groups of 0.82 log in the IG and 0.96 log in the CG, between study baseline and end-point, reached statistical and possibility clinical significance, since falls of greater than 0.5 log correlate with lower risk of disease progression [58].

The decline in viral load observed may have stemmed from small increases in adherence among patients, in conjunction with the potency of the therapeutic regimen [3]. Comparison of the evolution of viral load between the study participants and eligible individuals who refused to take part, showed decreases in both groups, albeit to a greater, statistically significant degree among participants of the clinical trial. This finding suggests that participation in the study in itself may have promoted an adherence-enhancing effect which in turn led to the differences in viral load detected between the two groups (participants of the study vs. refusals).

This study was the second randomized, controlled trial to increase ART adherence conducted in Brazil. As recommended by systematic reviews, adherence evaluation was objective, assessed in different points of the intervention and measured two months after its closure. Data was analyzed by intention to treatment and clinical results were also evaluated, an ethical requirement for these studies that aim at other benefits, as viral replication control [59, 60].

We sought to overcome some of the drawbacks of previous studies noted by other authors by running this study in a setting other than that of clinical efficacy trials [55, 56].

Lastly, it is important to emphasize that one of the contributions of this study was demonstrating the viability of running trials in health services by exploiting the potential of human resources and materials available, without introducing radical changes in the routines of professionals and patients [61].

## Limitations

The methodology originality and the short intervention period required by the funders demanded stricter control of its complexity. The venue option was the chosen reference and training center for an AIDS service that, compared to most Brazilian services, is better qualified and used as a benchmark. Given these constraints and limitations the present study did not benefit from the running of previous pilot studies, but may contribute to the design of future interventions in services which treat PLHIV in the State of São Paulo.

The experience of the health care team, the CRT-DST/AIDS facilities and its abundant and broad resources supported the study feasibility. In addition, the usual care provided by this service of technical excellence, offering a broad array of activities to encourage greater adherence, may have been an additional factor reducing the difference between the groups. Moreover, the Center’s differentiated structure and history may demand further adaptation of the intervention to other service contexts.

This study has several other potential limitations resulting from this context. First, it was not possible to recruit the number of volunteers initially envisaged for the two groups. The number of eligible subjects in the service was lower than expected and the quantity of refusals resulted in a smaller than recommended sample.

Another limitation was patient attrition of 20 % in the IG and 11 % in the CG. The higher attrition in the IG may be related to the same reasons for refusal reported by those who did not agree to participate, such as having to come more frequently than usual to the health service. The periodicity of every-15 days was not feasible for all patients as assumed, and should be considered as a relevant result in the adaptation in each service contexts. In the routine care, periodicity may be tailored to each person daily activities, to his/her working and domestic scenarios, to the long distances they have to cover to assess the services. Another paper will focus on process evaluation, in order to discuss how to respond to the social and daily life context of these patients who refused or abandoned the trial because of other important daily life activities. Expected differences (20 %) in adherence between the IG and CG was overestimated in the sample size calculation. The statistical power calculated post hoc did not exceed 12 %, and was therefore lower than the 80 % power used for sample size calculation.

## Conclusions

The intervention did not increase adherence among study participants. However, the reduced levels of viral load detected in both groups may have benefited the study participants because lower levels are associated with reduced mortality and disease progression.

Interventions aimed at increasing treatment adherence occur in the complex real daily life of health services, and this factor must be considered when using this framework or taking this structured intervention as an inspiration to other initiatives. The assistance provided through the intervention did not prove sufficient to impact adherence levels at this service. Indeed, we continue to believe that this population requires individualized attention that fosters each person’s participation as part of the solution, not as part of the problem, in line with the non-discrimination and participation principles of human rights-based initiatives.

Investing in the development of in-depth, individualized approaches aimed at promoting equity among individuals more vulnerable to non-adherence and AIDS morbidity, as well as at monitoring the Cuidado (Care) of patients with known adherence problems toward more effective solutions to tackle treatment problems, remains an ongoing challenge for health services.

Ongoing qualitative analysis of the intervention will yield further insights regarding the results of this pragmatic clinical trial. After refining the intervention, similar studies involving other less complex specialized services can be conducted in a range of different care settings which more closely reflect the prevailing reality of the national Unified Health System (SUS) and its Brazilian AIDS Response (BRA).

## Footnotes

1 For a deeper understanding of Brazilian Unified Health System see the Lancet The Brazil Series that critically examines what the country's policies have achieved and where future challenges might lie, accessible at http://www.thelancet.com/series.
